# 264. COVID-19 Associated Pulmonary Aspergillosis in Intensive Care Patients Based on Duration of Corticosteroid Administration

**DOI:** 10.1093/ofid/ofac492.342

**Published:** 2022-12-15

**Authors:** Meera D Shah, G Christina Gutierrez, Rebecca Moote, Kelly R Reveles, Elizabeth O Hand

**Affiliations:** University Health, San Antonio, Texas; University Health/University of Texas Health Science Center, helotes, Texas; University of Texas College of Pharmacy and University Hospital, San Antonio, Texas; The University of Texas at Austin, San Antonio, Texas; University of Texas Health Science Center San Antonio, San Antonio, Texas

## Abstract

**Background:**

Coronavirus disease 2019 (COVID-19) associated pulmonary aspergillosis (CAPA) has emerged as a complication in critically ill COVID-19 patients. Corticosteroids are standard of care for COVID-19 patients. Prolonged corticosteroids have been associated with an increased risk of CAPA, but duration of use associated with this risk remains unknown. The objective of this study was to evaluate if the duration of corticosteroid therapy ≤ 10 days vs > 10 days affects the risk of developing CAPA.

**Methods:**

This single-center, retrospective, cohort study included patients ≥ 18 years old admitted to University Hospital between March 2020 and December 2021, diagnosed with severe COVID-19 pneumonia requiring mechanical ventilation, and who received at least 3 days of corticosteroid treatment. CAPA was defined according to 2020 European Confederation of Medical Mycology and the International Society for Human and Animal Mycology consensus guideline. Baseline characteristics, CAPA, and secondary outcomes were compared using appropriate bivariable analyses. Steroid duration was evaluated as an independent predictor of CAPA in a logistic regression model.

**Results:**

Final analysis included 278 patients (n=169 ≤ 10 days steroid duration; n=109 > 10 day steroid duration). Baseline characteristics were similar between groups, with the exception of acute kidney injury and solid organ transplantation. Median duration of steroids was 6 days in the ≤ 10 day group vs 18 days in the > 10 day group. In total, 20/278 (7.2%) patients developed CAPA. Patients treated with > 10 days of corticosteroid therapy had significantly higher incidence of CAPA (12% vs 4.1%; p = 0.0156) and duration was independently associated with CAPA (OR 3.25, 95% CI 1.03-10.24). Secondary outcomes including inpatient mortality (43.2% vs 77.1%; p < 0.0001), mechanical ventilation free days at 28 (1.5 vs 0; p < 0.0001) and secondary infections (28.4% vs 44.9% p = 0.0220) were all significantly worse for > 10 day corticosteroid cohort.

**Conclusion:**

Duration of corticosteroid treatment > 10 days in critically ill patients is associated with an increased risk of CAPA. Though patients may require corticosteroids for non-COVID-19 indications, clinicians should attempt to limit prolonged courses of corticosteroids in COVID-19 patients.

**Disclosures:**

**All Authors**: No reported disclosures.

